# Genome-Wide Identification and Analysis of the Fatty Acid Export Family Revealed the Role of *GmFAX8* in Improving Soybean Oil Accumulation

**DOI:** 10.3390/plants14203166

**Published:** 2025-10-15

**Authors:** Yan Zhang, Yina Zhu, Xiuli Rui, Yuan Li, Jie Wang, Yuhang Zhan, Yongguang Li, Xue Zhao, Yingpeng Han, Xunchao Zhao

**Affiliations:** Key Laboratory of Soybean Biology in Chinese Ministry of Education (Key Laboratory of Soybean Biology and Breeding/Genetics of Chinese Agriculture Ministry), Northeast Agricultural University, Harbin 150030, China; zhangyan010122@163.com (Y.Z.); 15045389452@163.com (Y.Z.); rxl20000903@163.com (X.R.); liyuan02623@163.com (Y.L.);w15536351237@163.com (J.W.); zyhsoybean@163.com (Y.Z.); yongguangli@neau.edu.cn (Y.L.); xuezhao@neau.edu.cn (X.Z.)

**Keywords:** soybean, fatty acid transporters (FAX), gene family, oil accumulation

## Abstract

Fatty acid transporters (FAXs) play an important role in fatty acid synthesis by facilitating transport fatty acids from the plastid to the endoplasmic reticulum. This process is essential for providing precursor substances necessary for triglycerides (TAGs). Although *FAX* genes have been identified in variety of plant species, the identification and molecular functions of the *GmFAX* gene members in soybean are still unclear. In this study, soybean *FAX* genes were identified through the utilization of the Phytozome (v13) and NCBI online websites. Subsequently, phylogenetic trees, expression patterns, gene structures, and qRT-PCR were analyzed. A total of eight *GmFAX* members were identified at the whole genome level, and further phylogenetic analysis revealed that these members can be categorized into four subfamilies. In addition, all members of *GmFAX* contain a highly conserved domain Tmemb_14. Through qRT-PCR analysis, it was found that the expression level of the *GmFAX8* gene is relatively high in leaves and stems. Further investigation revealed that the total fatty acid content in hairy roots overexpressing the *GmFAX8* gene was significantly greater than that observed in the control strain. The results presented above suggest that the *GmFAX8* gene may play an important role in the accumulation of oil within soybeans.

## 1. Introduction

*Fatty acids* (FAs) are fundamental components of living organisms and are widely distributed in plants [1,2]. Fatty acids play a crucial role in the synthesis of triacylglycerols (TAGs), and serve as the primary component in oil biosynthesis [3,4]. The FA synthesis pathway represents one of the most essential metabolic pathways in organisms, characterized by its relative complexity [5]. The synthesis of fatty acids predominantly relies on acetyl-CoA as a substrate, which is derived from pyruvate produced through the glycolysis pathway from glucose [6]. Acetyl-CoA is condensed with malonyl-CoA to generate precursors for fatty acid synthesis. These precursors undergo a series of reduction, dehydration, and further reduction processes, ultimately resulting in the synthesis of long-chain fatty acids [5]. Then fatty acids are transported to their respective tissue sites via various transporters [7].

*FAX* serves as the initial transporter protein in the FA shuttle, and plays an important role in lipid synthesis [8]. The fatty acid transporter (FAX) gene is crucial for the regulation of lipid metabolism in plants, and it is widely present in animal and plant cells [8,9]. *FAX* genes play a crucial role in the transport of fatty acids from plastids to the endoplasmic reticulum, thereby supplying precursors necessary for triglyceride synthesis [10]. Previous studies have shown that *FAX* genes are crucial in the regulation of plant growth and development, as well as in the processes of oil synthesis [11]. So far, the *FAX* genes of many plant species have been cloned and characterized, including *Arabidopsis*, rapeseed, and green algae. In *Arabidopsis*, the loss of *FAX1* function disrupts intracellular fatty acid/lipid homeostasis, further damaging the formation of pollen walls and pollen viability, leading to male sterility [12]. Similarly, double mutants of *FAX2* and *FAX4* exhibit reduced FA export compared to the wild type, resulting in a significant decrease in TAG content in the seeds [13]. In green algae, *CrFAX1* and *CrFAX5* participate in TAG synthesis by functioning in chloroplasts and endoplasmic reticulum membranes, respectively. *CrFAX1* and *CrFAX5* synergistically shuttle FA from chloroplasts to endoplasmic reticulum for TAG synthesis [14]. The overexpression of the *FAX1-1* gene can significantly increase seed oil content in Brassica napus, and it was further found that the levels of PC and diacylglycerol (DAG) were significantly increased [15].

Soybean (*Glycine max*) is a major global oil crop, and increasing its seed oil content is a key objective for genetic improvement. Therefore, identifying key genes regulating soybean oil accumulation is crucial for developing high-oil varieties. Although *FAX* genes have been characterized in many plant species, the *FAX* gene family in soybean remains uncharacterized, and its role in regulating oil accumulation is unclear. In this study, eight *FAX* genes were identified in soybean, and their phylogenetic, gene structure, collinearity, and promoter elements were analyzed. In addition, it has also been preliminarily confirmed that the *GmFAX8* gene plays a role in enhancing oil accumulation in soybeans.

## 2. Results

### 2.1. Identification of GmFAX Genes in Soybean

**Figure 1:**
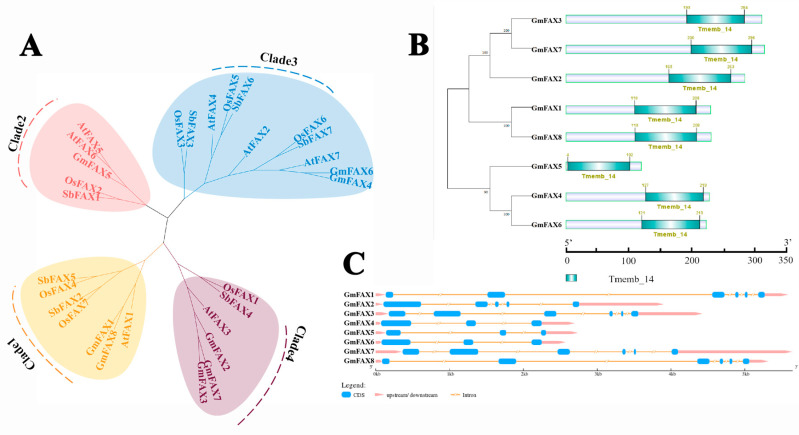
(**A**) Phylogenetic analysis of *FAX* proteins in soybean (*Glycine max*), sorghum (*Sorghum bicolor*), Arabidopsis (*Arabidopsis thaliana*), and rice (*Oryza sativa*) was performed using MEGA11. The resulting phylogenetic tree revealed four distinct subfamilies, visually represented by concentric colored rings surrounding the terminal branches: subfamily I (yellow), subfamily II (pink), subfamily III (blue), and subfamily IV (purple). (**B**) Conserved domain architecture analysis of *GmFAX* proteins in soybean. The schematic representation highlights a signature domain (green) shared across all *GmFAX* family members, annotated using Pfam database criteria. Study on the structure and function of *GmFAX* genes in soybean. (**C**) The schematic diagram shows the exon intron region of soybean *GmFAX* genes, and the untranslated region (UTR), exon, and intron are represented by pink, blue, and orange respectively.

To identify the *FAX* gene family members in soybean, the protein sequence of *Arabidopsis FAXs* was used as queries to perform a BLASTP search against the soybean genome in the Phytozome (v13) database. A total of eight *GmFAX* genes were identified and named *FAX–FAX8* based on their chromosomal positions. The full-length CDS sequence of *GmFAX1–GmFAX8* ranged from 360 bp to 951 bp. The predicted isoelectric point of GmFAX genes ranged from 5.68 to 9.8, and their molecular weight ranged from 12.57 to 34.57 kDa. In order to further understand the phylogenetic relationship of the soybean *FAX* gene family, the *FAX* proteins of *Arabidopsis*, *Glycine max*, *Oryza sativa,* and *Sorghum bicolor* were obtained from the Phytozome (v13), and the phylogenetic tree was generated through MAGE version 11.0 software. The phylogenetic tree revealed that the *FAX* genes are divided into four distinct subfamilies (I–IV). Subfamily I includes *GmFAX1* and *GmFAX8*; subfamily II contains *GmFAX5*; subfamily III comprises *GmFAX4* and *GmFAX6*; and subfamily IV consists of *GmFAX2*, *GmFAX3*, and *GmFAX7*. To explore the gene structure of *FAX* genes, the gene structure of *GmFAX* genes was generated through GSDS online website. As shown in Figure 1, members of subfamily I typically possess six exons, whereas genes in subfamilies II and III contain only three exons. Conserved domain analysis performed with the SMART database revealed that all *GmFAX* proteins contain the Tmemb_14 domain, suggesting that their functions are evolutionarily conserved (Figure 1).

### 2.2. Collinearity Analysis of GmFAXs

**Figure 2:**
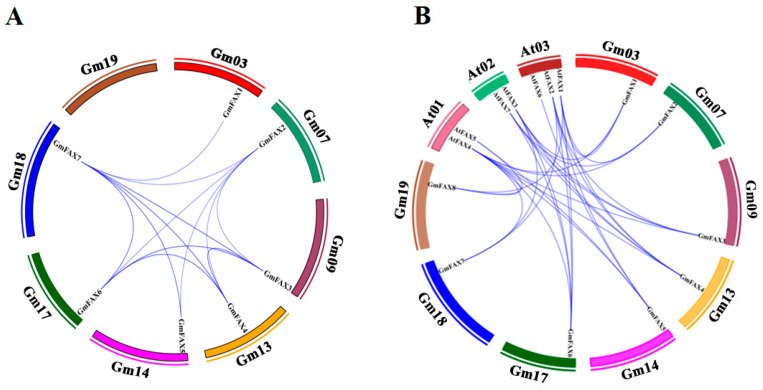
(**A**) Genomic distribution and duplication events of *GmFAX* loci across soybean chromosomes, with segmentally duplicated pairs connected by blue arcs. (**B**) Cross-species syntenic relationships of *FAX* homologs between *Glycine max* (soybean) and *Arabidopsis thaliana,* as determined by TBtools version 2.316.

To further characterize duplication events within the soybean genome, a synteny analysis of *GmFAX* genes was performed. The eight *GmFAX* genes were located on eight different chromosomes (Chr03, 07, 09, 13, 14, 17, 18, and 19). The synteny analysis identified 20 duplicated gene pairs among these *GmFAX* genes (Figure 2). A total of 20 pairs of syntenic gene pairs were identified in soybean. Furthermore, to trace the evolutionary history of these genes, we conducted a comparative syntenic analysis between soybean and *Arabidopsis thaliana*. As shown in Figure 2, there are duplicate events between *GmFAXs* and *AtFAXs*, including 30 pairs of reciprocal relationships between *G. max* and *A. thaliana*.

### 2.3. Analysis of Cis-Acting Elements of GmFAX Promoter

**Figure 3:**
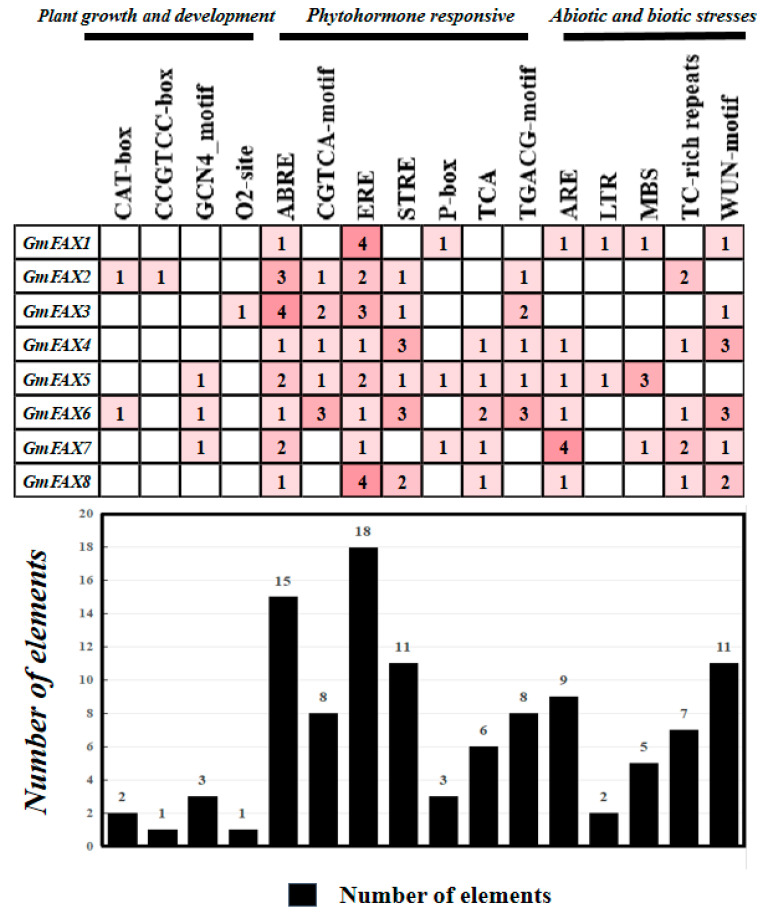
Analysis of cis-elements in the promoter of *FAX* genes. Number of cis-elements in the 2.0 kb promoter region upstream of *GmFAX* genes. The black box corresponds to the total number of cis-elements.

To further explore the cis-acting elements of the *GmFAX* genes promoter, the 2 kb genomic sequence upstream of the translational start site (ATG) of each *GmFAX* gene was analyzed. As shown in Figure 3, ABRE (abscisic acid-responsive element) and ERE elements were found in all *GmFAXs*. CAT-box (involved in meristem expression) elements were found in *GmFAX2* and *GmFAX6*. The CCGTCC box element was only found in *GmFAX2*. Notably, an LTR (low temperature responsiveness) element was identified in *GmFAX1* and *GmFAX5*, while an MBS (drought inducibility) element was found in *GmFAX1*, *GmFAX5*, and *GmFAX7*. The O2 site element was only found in *GmFAX3* (Table S4). The prevalence of these stress- and hormone-related cis-elements strongly suggests that *GmFAX* genes play important roles in mediating plant growth, development, and responses to environmental stimuli.

### 2.4. Expression Pattern of Soybean FAXs in Different Developmental Stages

**Figure 4:**
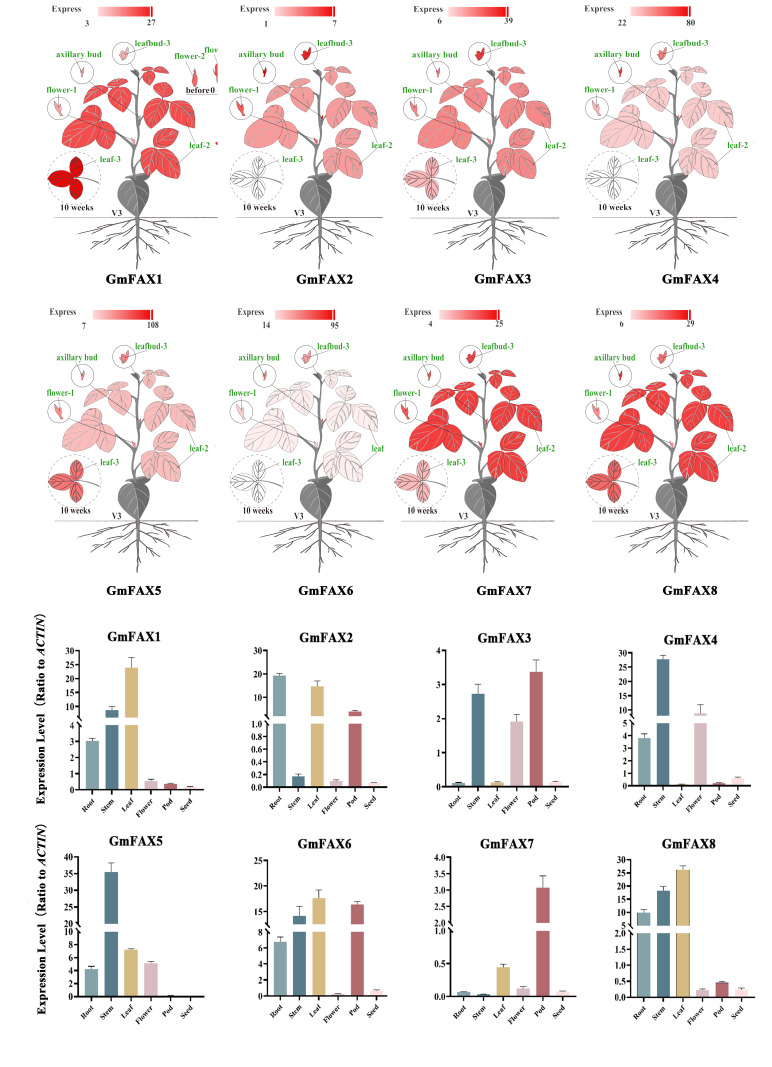
Expression patterns of *GmFAXs* in different tissues.

In order to determine the expression pattern of soybean *FAX* genes at different developmental stages, the high-throughput sequencing data of the National Bioinformatics Center (https://ngdc.cncb.ac.cn/soyomics/transcriptome/) (accessed on 27 March 2025) were retrieved and analyzed. As shown in Figure 4, the expression of *GmFAX* genes was investigated in different tissues. *GmFAX4* was highly expressed in leaves, seeds, and root nodules, while *GmFAX5* and *GmFAX8* showed broad expression patterns across multiple tissues. At the same time, it was found that the expression levels of *GmFAX5* and *GmFAX8* genes were the highest in the flowers. In contrast, *GmFAX2* displayed universally low expression levels across all examined tissues. Subsequently, through qRT-PCR, we found that the expression level of *GmFAX2* was relatively low in different tissues, while the expression levels of *FAX1* and *FAX8* genes were relatively high in the roots, stems, and leaves. The expression levels of *FAX4* and *FAX5* were relatively high in the stems (Figure 4).

### 2.5. Subcellular Localization of GmFAX8

**Figure 5:**
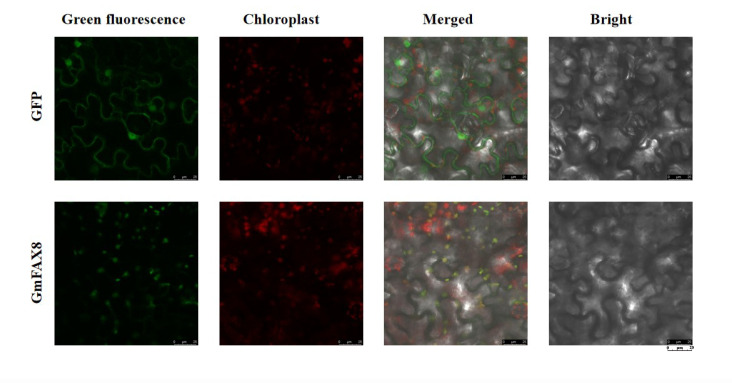
Subcellular localization analysis of *GmFAX8*.

The *GmFAX8* gene was amplified from the DN50 cDNA, and then it was constructed into the pCAMBIA3300 vector containing green fluorescent protein (GFP) and transformed into GV3101 (pSoup-p19) competent cells. The recombinant 35S::*GmFAX8*-GFP plasmid, along with the positive control (35S::GFP) was transferred into tender Nicotiana tabacum using the *Agrobacterium* mediated method. As shown in Figure 5, the tobacco leaves injected with the pCAMBIA3300-GmFAX8-GFP expression vector showed only visible green fluorescence at the chloroplasts when observed under a laser confocal microscope. No nuclear or cell membrane expression was found. The empty pCAMBIA3300-GFP could be observed to have green fluorescence at the cell membrane, cytoplasm, and nucleus, indicating that the *GmFAX8* protein is mainly localized in the chloroplasts.

### 2.6. Overexpression of GmFAX8 Increased Total Fatty Acid Content

**Figure 6:**
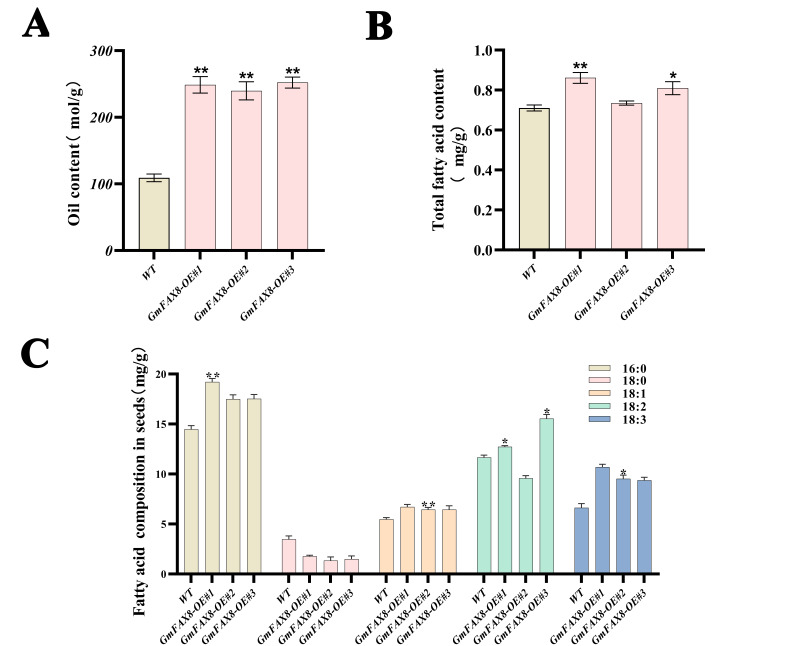
(**A**). TAG content in Saccharomyces cerevisiae. (**B**). Total fatty acid content in hairy root of soybean. (**C**) The fatty acid composition in seeds. * indicates significance at *p* < 0.05. ** indicates significance at *p* < 0.01.

To investigate the role of *GmFAX8* in total fatty acid accumulation, its coding sequence was cloned into the pYES3 vector, and the recombinant vector was transformed into *Saccharomyces cerevisiae* strain INVSc1. The oil content in yeast cells expressing the pYES3-GmFAX8 construct was significantly higher than in those harboring the empty pYES3 vector, which indicated that the *GmFAX8* gene promotes the accumulation of oil content. Consistent with the yeast results, the total fatty acid content in transgenic *GmFAX8* hairy roots was also significantly higher than in control lines (Figure 6), as determined by gas chromatography. Subsequently, when analyzing the contents of each component of fatty acids, we found that the contents of palmitic acid, oleic acid, linoleic acid, and linolenic acid were significantly higher than those of the control group. However, the content of stearic acid was significantly lower than that of the control group.

### 2.7. Co-Expression Analysis of Transcription Factors and GmFAXs in Soybean

**Figure 7:**
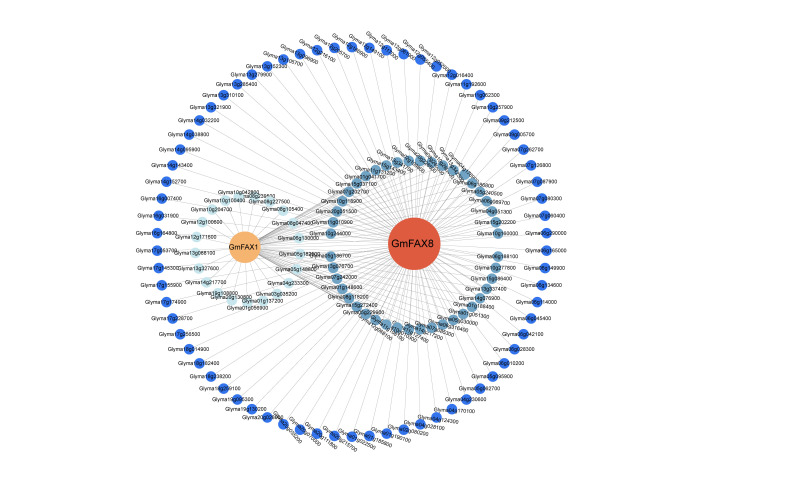
Co-expression network of transcription factors and soybean *FAXs*.

In order to further clarify the regulatory mechanism of upstream transcription factors and *GmFAX* family genes at the transcription level, we constructed a co-expression network with transcription factors and *GmFAX* genes. As shown in Figure 7, a total of 179 co-expression modules were constructed, encompassing *GmFAX1*, *GmFAX8*, and 136 transcription factors. *GmFAX8* showed a significant positive correlation with *Glyma13g341700*, *Glyma11g131200*, *Glyma10g160000*, *Glyma06g042100*, *Glyma15g086400,* and *Glyma18g014900* (r > 0.90, *p* < 0.05). *GmFAX1* showed a significant positive correlation with *Glyma10g160000* (r > 0.97, *p* < 0.05). It is noteworthy that both *GmFAX8* and *GmFAX1* showed a significant positive correlation with the *Glyma10g160000* gene (r > 0.90, *p* < 0.05). The above results suggest that the *GmFAX8* gene may interact with multiple transcription factors to further regulate the accumulation of lipids.

### 2.8. Haplotype Analysis of the GmFAX8 Gene

**Figure 8:**
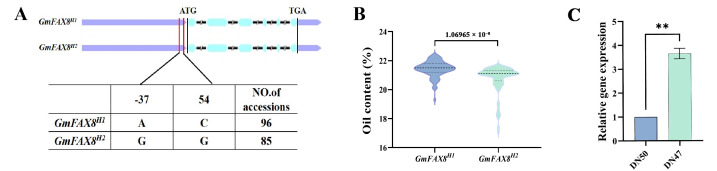
(**A**)* GmFAX8* gene promoter (2.0 kb) and gene region haplotype detection. (**B**) Comparative analysis of the oil content of the three haplotypes. (**C**) The relative expression levels of the *GmFAX8* gene in high-oil varieties and low-oil varieties. ** indicates significance at *p* < 0.01.

In order to investigate the relationship between the *GmFAX8* gene and the oil content of soybeans, we conducted a gene-based association analysis using the GLM model. In 181 soybean samples, two SNPs significantly related to the oil content were identified, namely rs39595759 and 39,595,813 (−log10 (P) ≥ 2.5) [10], which were located in the 5′ UTR and upstream regions of the *GmFAX8* gene, respectively (Figure 8A). The result analysis revealed that the oil content of haplotype 1 was significantly higher than that of haplotype 2 (Figure 8B). Further analysis revealed that the expression level of the *GmFAX8* gene in high-oil materials was significantly higher than that in low-oil materials (Figure 8C).

## 3. Discussion

*FAX* genes play an important role in the FA pathway and are mainly involved in the transmembrane transport of fatty acids [16]. Previous studies have shown that *FAXs* play an important role in the accumulation of seed oil across various species [8,16]. Although *FAX* genes have been analyzed in various plant species, including *Arabidopsis* [13], *Brassica napus* [17], *Camelina sativa* [18], and *Green microalga* [14], their identification and molecular functions in soybean remain unclear. It has been reported that *FAX* plays a crucial role in the transport of fatty acids, but the transport of fatty acids in soybeans still needs further exploration. In this study, eight *GmFAX* genes were identified in the soybean genome. Phylogenetic analysis can clearly prove the evolutionary relationship between soybean *FAX* and other species. The results showed that the eight *GmFAXs* were categorized into four distinct clusters according to their different evolutionary relationships and functional characteristics. All GmFAXs contained Tmemb_14 conserved domains. These results indicate that *GmFAX* genes were relatively conserved. Previous studies have identified seven *AtFAX* genes in *Arabidopsis thaliana*, named *AtFAX1–7*, which were divided into seven sub-clusters and performed different functions [8]. For instance, *AtFAX1* (subfamily I) is localized to the chloroplast inner membrane of chloroplast and mediates the output of fatty acids from chloroplast [15]. Subfamilies II and IV, *AtFAX2* and *AtFAX4,* are able to transport fatty acids into the endoplasmic reticulum, where they can be further converted into triacylglycerols [13]. Currently, the function of the *FAX* genes has been found in various species, including *Arabidopsis*, rapeseed, and green algae [16,17]. It was found that *GmFAX* genes are expressed in different tissues through the online website of the National Biological Information Center. The expression levels of *GmFAX4*, *GmFAX5,* and *GmFAX6* were higher in different tissues, while the expression levels of *GmFAX2* were lower in different tissues. Subsequently, the expression level of *GmFAXs* in different tissues was analyzed via qRT-PCR. It was found that the expression level of *GmFAXs* in stems and leaves was relatively high. Previous studies have found that *AtFAX2* and *AtFAX4* are highly expressed in the roots, stems, and leaves of *Arabidopsis thaliana* [13]. In rapeseed, *FAX1* also has a relatively high expression level at different stages of tissue development. [17]. Phylogenetic tree and domain analysis indicated that *FAX1* and *FAX8* had high homology. Further co-expression network analysis revealed that transcription factors were positively correlated with *FAX1* and *FAX8,* respectively. The above results indicate that the *FAX1* gene may have a positive effect on lipid accumulation. To further elucidate the molecular function of *GmFAX8* in lipid accumulation, transgenic soybean hairy roots were generated. The results showed that the total fatty acid content in *GmFAX8*-overexpressing hairy roots was significantly higher than in the control lines. Previous studies have found that the overexpression of *BnaA09-FAX1* in Brassica napus can significantly increase the content of PC and DAG [17]. In Chlamydomonas reinhardtii, the co-expression of *FAX1* and *FAX2* significantly increased the content of triacylglycerol (TAG) [19,20,21]. In rapeseed, the overexpression of *BnaFAX1-1* significantly increased the content of oleic acid (C18:1) in seeds [15]. Previous studies have found that the *bHLH7* gene in *Arabidopsis thaliana* can significantly increase the oil content of seeds [22]. In this study, through the co-expression network, *bHLH* (*Glyma13g341700*) was identified to have a significant positive correlation with *GmFAX8*, which suggests that this gene plays an important role in regulating lipid accumulation. Through candidate gene association analysis, this study identified two haplotypes. Among them, haplotype 1 had a significantly higher oil content than haplotype 2. The above results can provide a basis for molecular-assisted selection breeding.

In conclusion, a total of eight soybean *FAX* genes were classified into four distinct clusters. These *GmFAX* genes exhibited varying expression levels at different developmental stages, and it was preliminarily demonstrated that the *GmFAX8* gene can regulate the accumulation of soybean oil.

## 4. Materials and Methods

### 4.1. Identification of the FAX Gene Family

In order to screen the members of the *FAX* gene family in soybean, the reported protein sequences of *Arabidopsis FAX* family members were retrieved from the TAIR and used as alignment sequences to perform BLASTP sequence alignment with the soybean genome database on the online website of Phytozome (v13) (https://phytozome-next.jgi.doe.gov/). Candidate genes were selected based on an E-value < 10^−10^ and sequence similarity >90% [23]. The presence of the characteristic FAX domain in these candidates was further verified using the SMART and Pfam databases [24,25]. The candidate genes of the soybean *FAX* gene family were determined, and their domain distribution map was drawn using IBS 1.0 [26]. The physicochemical properties of soybean *GmFAX* family members, including molecular formula, protein molecular weight, and isoelectric point, were predicted through the bioinformatics resource website ExPASy (http://www.expasy.org) [27].

### 4.2. Phylogeny, Genetic Structure, and Collinearity Analysis of the FAX Gene Family

The FAX protein sequences of soybean (*Glycine max*), Arabidopsis thaliana (*A. thaliana*), rice (*O. sativa*), and sorghum (*Sorghum bicolor*) were used to construct a phylogenetic tree based on the neighbor joining method and bootstrap test set at 1000 replicates using MEGA11 [28]. The intron/exon position structure of the *FAX* genes was obtained through the GSDS (https://gsds.gao-lab.org/Gsds_help.php) (accessed on 17 March 2025) online website [29]. To investigate the evolutionary relationships between the *FAX* genes of soybean and *Arabidopsis*, a collinearity analysis was performed using TBtools software [30].

### 4.3. Promoter Analysis of GmFAXs

In order to explore the key cis-acting elements in the upstream promoter region of *GmFAX* genes, the 2 kb upstream promoter sequence of *GmFAX* genes were obtained from Phytozome v13 database (https://phytozome-next.jgi.doe.gov/), and submitted to the PlantCARE database to analyze the category, quantity, and location of the cis-acting elements [23].

### 4.4. Expression Analysis of GmFAX During Soybean Development

The expression levels of the *GmFAX* gene in soybean at different developmental stages were obtained through the National Bioinformatics Center database (https://ngdc.cncb.ac.cn/soyomics/index)(accessed on 25 March 2025). To further confirm the expression levels of the *GmFAX* gene during different developmental stages, soybean seeds (DN50) were used as materials, and three plants with consistent growth were selected to collect samples at different developmental stages, including roots, stems, leaves, flowers, pods, and seeds. The collected samples were rapidly frozen in liquid nitrogen, and then RNA was extracted from each sample using Trizol reagent (Takara, Beijing, China). Quantitative real-time qRT-PCR was performed using the CFX Connect TM real-time system (Bio-RAD, Hercules, CA, USA) and SYBR Select Master Mix qRT-PCR (SYBR Green, Toyobo, Osaka, Japan). GmActin4 (GenBank accession number AF049106) was used as the internal reference. The expression level of *GmFAX* genes was calculated by 2^−ΔΔCt^ [31]. Each reaction was set up with three biological replicates and three technical replicates.

### 4.5. Subcellular Localization Analysis of GmFAX8

Using the cDNA of soybean DN50 as the template, the CDS region of *GmFAX8* gene was amplified and cloned into the pCAMBIA3300 vector, which harbored a green fluorescent protein (GFP) tag to generate the pCAMBIA3300-GmFAX8::GFP construct to generate pCAMBIA3300-*GmFAX8*::GFP. The recombinant plasmid pCAMBIA3300-*GmFAX8*::GFP and the empty plasmid pCAMBIA3300 were transformed into *Agrobacterium* GV3101 (pSoup-p19), and the young leaves of tobacco were transiently infected by *Agrobacterium*. The empty vector pCAMBIA3300 (containing green fluorescent GFP label) was used as the control. The expression of the pCAMBIA3300-*GmFAX8*::GFP fusion protein was observed by a laser confocal microscope (laser confocal microscope, SP8 LIGHTNING, Wetzlar, Germany).

### 4.6. Overexpression of GmFAX8 in Soybean Hairy Roots

The above-mentioned recombinant plasmid (pCAMBIA3300-GmFAX8::GFP) and empty pCAMBIA3300 plasmid were transferred into *Agrobacterium rhizogenes* K599, respectively. Soybean (DN50) was introduced using the *Agrobacterium rhizogenes*-mediated cotyledonary node method. The growth of hairy roots was observed by explant preparation, infection, co-culture, and rooting. The culture dish was taken out at the right time, and the residual culture medium was rinsed with distilled water for PCR detection to obtain transgenic hairy roots [32].

### 4.7. Determination of Total Fatty Acid Content Using the Gas Phase Method

In this experiment, gas chromatography (GC) was employed to measure the fatty acid content in the hairy roots of soybeans. We plotted a standard curve using five major fatty acids as internal standards, with the concentration unit of the internal standards being milligrams per milliliter. The *x*-axis of the standard curve represented the concentration (milligrams per milliliter), and the *y*-axis represented the peak area. After drying the above-mentioned transgenic hair-like roots, 0.1 g was weighed, ground, and added to 3 mL of n-hexane. It was extracted in a 50-degree water bath for 1 h (mixed up and down once every 5 min). After extraction, 3 mL of sodium hydroxide methanol solution (0.5 mol/L) was added, and the vortex was shaken for 30 min. It was left to stand at room temperature for 60 min. The supernatant was filtered into a 1.5 mL brown bottle. The fatty acid content was then determined using an Agilent gas chromatograph. Meteorological chromatographic conditions were as follows: quartz capillary column; the carrier gas was helium; the initial temperature of the column was set at 180 °C and kept for 1.5 min; the temperature was raised to 225 °C at 10 °C/min and kept for 2 min; the injection port temperature was set at 250 °C, and the column flow rate was 67.5 mL/min; and the split ratio was set as 20:1. All data were subjected to three biological replicates.

### 4.8. Determination of Triglyceride Content in Yeast

The amplified *GmFAX8* gene was connected to the vector pYES3 to obtain the recombinant vector pYES3-*GmFAX8*. The recombinant plasmids pYES3-*GmFAX8* and pYES3 were transferred into Saccharomyces cerevisiae INVSc1. The yeast was grown in a solid SD-Trp medium (containing 2% galactose) and cultured at 30 °C for 48 h. The content of TG and protein in the recombinant vector and empty vector (pYES3) was determined by the enzyme assay kit for the content of triglycerides (TGs) in tissue cells (Applygen Technologies Inc., Beijing, China) and the BCA protein quantitative Kit (Applygen Technologies Inc., Beijing, China).

### 4.9. Co-Expression Network Analysis of GmFAX Genes and Transcription Factors

To investigate the co-expression relationships between transcription factors and the target genes, we utilized transcriptome data from 10 soybean varieties (5 high-oil and 5 low-oil) at the R6 developmental stage [33]. Differentially expressed genes (DEGs) were first identified with a threshold of |Log2FC| > 0.6. Transcription factors and their potential target genes among these DEGs were then selected for network analysis. The co-expression network relationships were constructed using the R package version 4.4.1 (|r ≥ 0.75|, *p* < 0.01).

### 4.10. Haplotype Analysis of the GmFAX8 Gene

The whole genome of 181 different varieties of soybean varieties was re-sequenced at 30× coverage. With MAF > 0.1 as the threshold for filtering, 1,355,928 SNP markers were obtained. The general linear model (GLM) of TASSEL 5.0 software was used to analyze the sequence variations of the *GmFAX8* gene based on the phenotypic values of oil content. SNPs with a threshold of −log10 (P) ≥ 2.5 were defined as significantly associated loci. The single-nucleotide polymorphisms of the gene promoter, 5′ untranslated region, exon, and 3′ untranslated region of *GmFAX8* were studied.

## Data Availability

The original contributions presented in this study are included in the article/supplementary material. Further inquiries can be directed to the corresponding authors.

## Supplementary Materials

The following supporting information can be downloaded at: https://www.mdpi.com/article/10.3390/plants14203166/s1, Table S1: Primers used for overexpression and qRT-PCR. Table S2: Basic information of the eight soybean FAX genes. Table S3: Co-expression network correlation coefficient. Table S4. The cis-acting elements and locations in the promoter region of the FAX gene family.
