# Supplementary material for: Genome-Wide Identification and Analysis of the Fatty Acid Export Family Revealed the Role of GmFAX8 in Improving Soybean Oil Accumulation
Source: Plants (Basel). 2025 Oct 15;14(20):3166. doi: 10.3390/plants14203166 (PMC12567294; doi:10.3390/plants14203166)
Supplement: Supplementary file 1 [file plants-14-03166-s001.zip › TableS2.pdf]

Table S2 Basic information of the sixteen soybean *FAX* genes.

Gene name	Gene ID[1]	Direction	ORF[2] length (bp)	Isoelectric point	Molecular Weight (KDa)
FAX1	*Glyma.03G133200*	forward	693	9.74	24.6
FAX2	*Glyma.07G108500*	reverse	858	5.93	31.4
FAX3	*Glyma.09G274500*	reverse	939	5.68	34.1
FAX4	*Glyma.13G116300*	reverse	687	9.52	22.5
FAX5	*Glyma.14G024900*	forward	360	9.2	12.5
FAX6	*Glyma.17G043500*	forward	669	9.19	22.3
FAX7	*Glyma.18G213500*	reverse	951	6.77	34.5
FAX8	*Glyma.19G135000*	forward	693	9.8	24.7

[1]IDs are available in the soybean genome sequencing project database.

[2]ORF: open reading frame.
